# The Role of Superior Vena Cava Isolation in the Management of Atrial Fibrillation

**DOI:** 10.19102/icrm.2017.080406

**Published:** 2017-04-15

**Authors:** Rajat Goyal, Ely Gracia, Roger Fan

**Affiliations:** ^1^Department of Cardiology, Stony Brook University Hospital, Stony Brook, NY; ^2^Department of Internal Medicine, Stony Brook University Hospital, Stony Brook, NY; ^3^Heart Rhythm Center, Stony Brook University Hospital, Stony Brook, NY

**Keywords:** Atrial fibrillation, catheter ablation, superior vena cava, vena cava

## Abstract

The superior vena cava (SVC) has been identified as one of the most common sources of non-pulmonary vein triggers for atrial fibrillation (AF). SVC isolation has been shown to improve long-term maintenance of normal sinus rhythm in patients with paroxysmal AF. However, ablation at the SVC is associated with risks of phrenic nerve injury, sinus node dysfunction, and SVC stenosis. The use of electroanatomical mapping, intracardiac echocardiography, compound motor action potentials, and segmental (rather than circumferential) ablation are all strategies to reduce complications. Given these risks, SVC isolation is most effective as an adjunct to pulmonary vein isolation for patients with paroxysmal AF who have been found to have an arrhythmogenic SVC.

## Introduction

Catheter ablation of atrial fibrillation (AF), the most common sustained arrhythmia in the world, is an effective treatment modality for individuals with drug-resistant, symptomatic disease. Since it was first described by Haïssaguerre et al. in 1998, pulmonary vein isolation (PVI) has been a mainstay in the approach for ablation of AF.^1^ However, focal ectopic discharges arising from outside the pulmonary veins have also been shown to precipitate AF in up to 28% of patients.^2^ These can originate from multiple sites, including the ligament of Marshall, the coronary sinus, crista terminalis, posterior left atrium (LA), interatrial septum, left atrial appendage, or superior vena cava (SVC).^3^ The SVC is one of the most common non-pulmonary vein triggers of AF, and is therefore an attractive target for ablation in order to maintain normal sinus rhythm.^2,4^

### Etiology of arrhythmogenicity

Embryologically, the SVC arises from a communication between the sinus venosus and the right atrium. Structures that originate from the sinus venosus have demonstrated the ability to express cells that retain ectopic pacing abilities, and can become a focus of arrhythmia.^5^ These specialized cells have been found to reside within large atrial myocardial extensions (or myocardial sleeves) on the SVC, and serve as independent triggers for arrhythmias.^6^ These myocardial sleeves extend an average of 13.7 mm, but can be up to 47 mm long, into the SVC, with a mean thickness of 1.2 mm.^6^

Interestingly enough, it has been shown that pulmonary veins also exhibit myocardial sleeves. These sleeves are the result of the migration of atrial tissue into the pulmonary veins. As such, the myocardial sleeves over the pulmonary veins exhibit properties of rapid atrial conduction and some automaticity, facilitating the generation of atrial fibrillation.^7^ The myocardial sleeves over the SVC, however, exhibit properties similar to that of the sinoatrial node (SAN), in that they lack the expression of rapidly conducting gap junctions, but have a stronger propensity for automatically owing to the presence of Phase 4 depolarization, in addition to triggered activity.^7,8^

As a result, the predominant role of the SVC in AF appears to be as a trigger for AF, with triggers more likely to be found in larger and longer (i.e. more than 30 mm) sleeves.^9,10^ However, the SVC musculature may also be involved in sustaining AF.^11^ In a series of 74 patients with SVC-associated AF, Miyazaki et al. observed that in the majority of the cases (78.4%), the SVC was found to be a trigger of AF, in that AF originated from the SVC. Furthermore, once AF persisted, the AF cycle length was longer in the SVC than in the right atrium (RA), and SVC isolation (SVCI) did not result in AF termination. However, following SVCI, AF could no longer be induced.^11^ In 32.4% of patients, the SVC appeared to be a driver, or perpetuator, of AF. In these cases, AF cycle length in the SVC was the same or shorter than that of the RA, and AF was terminated or converted to atrial flutter by SVCI. In some of these individuals, AF was confined to the SVC following electrical isolation, proving that the SVC musculature is capable of sustaining AF.^11^

Arrhythmias arising from the SVC are also more sensitive to changes in parasympathetic tone. Specifically, increases in parasympathetic tone result in a shorter effective refractory period (ERP), which contributes to the increased risk of arrhythmia. Lu et al. demonstrated that high-frequency stimulation of the SVC-aorta ganglionated plexus in dogs precipitated AF within the SVC.^12^ Ablation of the plexus increased the baseline ERP and resulted in a lack of AF inducibility. This may serve as one explanation as to why obesity and obstructive sleep apnea can increase the risk of AF.^12^

### Its role in AF

The SVC is one of the most common sources of non-pulmonary-vein-related paroxysmal AF, found in 40% of patients with AF caused by non-pulmonary vein triggers.^13^ SVC triggers, when identified, are rarely found spontaneously. In the majority of cases, they are found following isoproterenol infusion, adenosine injections and/or cardioversion of AF.^2,11^

The involvement of the SVC in paroxysmal AF has been fairly consistent across multiple studies. Tsai et al.^4^ reported a 6% incidence of SVC-triggered paroxysmal AF in 130 patients, while Lee et al. reported 12.9% in 293 patients.^13^ Most recently, Zhao et al. documented a 7.4% incidence of SVC-triggered AF in 121 patients with paroxysmal AF and low ejection fraction (EF), with a similar 9.7% incidence in 140 patients with paroxysmal AF and normal EF.^14^

The role of the SVC in persistent AF is less well defined. Arruda et al. documented a 12% incidence of SVC triggers in 190 patients in a population that included 10% persistent AF and 39% permanent AF.^15^ In contrast, in a large series of 836 patients undergoing AF ablation, Miyazaki et al. found a significant difference in arrhythmogenic SVC prevalence between patients with paroxysmal AF (8.5%), persistent AF (1.9%), and long-standing persistent AF (1.3%).^11^ These authors found that an arrhythmogenic SVC was associated with fewer clinical predictors of AF progression. Patients with arrhythmogenic SVC were typically younger and less obese with a smaller LA, less hypertension, and less structural heart disease. Supporting this, in a recent study of 102 patients with long-standing persistent AF, the SVC was confirmed to be involved in the initiation and/or maintenance of AF in only one patient (0.98%).^16^

### Efficacy of SVC ablation in reducing recurrence of atrial fibrillation

Initial studies addressing the efficacy of ablation of non-pulmonary vein triggers discovered that they were amenable to ablation, with good one-year outcomes.^2,4^ Chang et al. found that in patients with AF who only had SVC triggers during electrophysiology study, SVCI without PVI could achieve freedom-from-AF rates of 73% at 5 years after a single procedure.^17^ These findings support the role of SVCI as an effective ablation strategy for managing patients with SVC trigger-induced atrial fibrillation.

Several randomized controlled trials evaluated the utility of empiric SVCI in addition to PVI for patients with AF. Corrada et al. randomized 320 individuals with paroxysmal, persistent, or permanent AF to PVI alone versus PVI with SVCI. Although there were no statistically significant differences in AF recurrence at 1 year between the two treatment groups, only the subset of patients with paroxysmal AF had a statistically significant higher chance of maintaining sinus rhythm (i.e. 77% following PVI versus 90% following PVI with SVCI).^18^ Similarly Ejma et al. evaluated 186 patients with paroxysmal AF in a non-randomized study, allocating the first half to undergo PVI + SVCI if triggers were present, and allocating the second half to undergo PVI + empiric SVCI. SVCI was performed in 9% of patients in the first group as a result of the identification of trigger or rapid SVC activity. During a mean follow-up period of 27 months, those who received empiric SVCI had a statistically significantly lower atrial tachyarrhythmia recurrence rate (44% versus 23%).^19^

Contrary to these findings, two other randomized controlled trials failed to show any significant benefit with regards to empiric SVCI. Wang et al. randomized 106 individuals with paroxysmal AF to either PVI or PVI + SVCI and found that there was no statistically significant difference in freedom from atrial tachyarrhythmia recurrence at 12 months (92.6% PVI versus 94.2% PVI + SVCI).^20^ Da Costa et al. also randomized 100 patients with paroxysmal AF to PVI or PVI + SVCI, and demonstrated that at long-term follow-up (15 ± 8 months), there was no statistically significant difference in atrial arrhythmias (18% versus 12%).^21^ However, the significance of both of these studies is limited by their small sample sizes and short follow-up periods. Also, notably, during the follow-up period in the study by Wang et al., 16% of all cases underwent redo ablation procedures. Almost all of the cases had pulmonary vein reconnections, yet none in the SVCI group demonstrated SVC reconnections. The only two patients in the PVI group without pulmonary vein reconnections were both found to have SVC tachyar-rhythmias, requiring SVCI. Hence, the small sample size and low prevalence of SVI triggers may have contributed to the negative outcomes.

Lastly, as mentioned earlier, the rarity of SVC arrhythmogenicity contributing to persistent AF was demonstrated by Xu et al., who found that only one out of 102 patients demonstrated a SVC trigger. Thus, these researchers concluded that in patients with long-standing persistent AF, empiric SVCI is likely to be unneccessary.^16^

### Technical approach to SVCI

**Figure 1:**
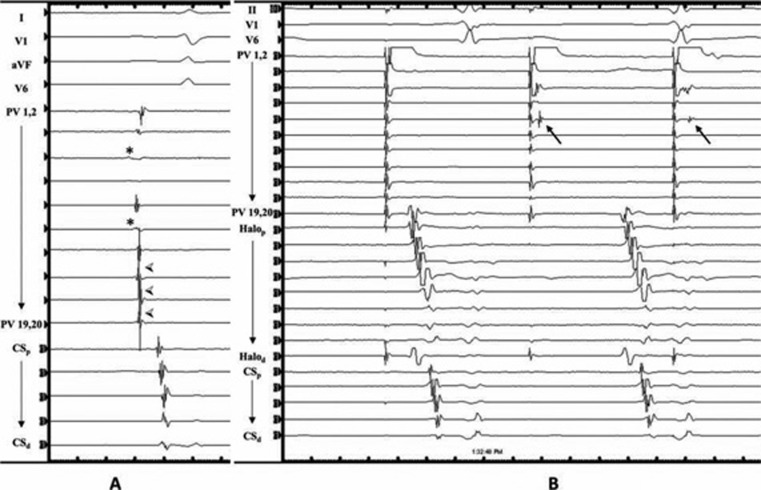
(a) Multi-electrode electrograms inside the superior vena cava (SVC) junction with far-field right atrium potentials (*) and sharp SVC sleeve potentials 
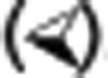
. Following SVC isolation, exit block can be seen in (b), with SVC pacing resulting in transient myocardial sleeve capture (arrows), and dissociation from the atrium.

**Figure 2:**
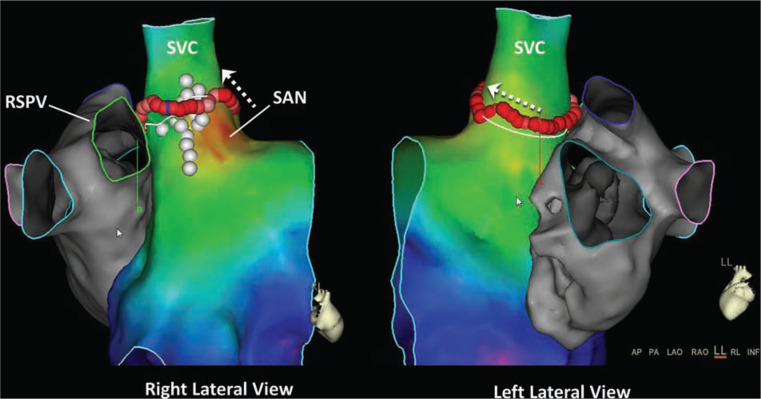
Sinus rhythm activation map. The white dots represent phrenic nerve capture. Note the proximity of the right superior pulmonary vein to the superior vena cava (SVC), and the location of the sinoatrial node (SAN) at the anterolateral SVC-RA (right atrium) junction. Circumferential ablation of the SVC requires more cranially placed lesions at the lateral SVC to avoid SAN injury (arrows).

SVCI is performed by placing a circular or multi-electrode mapping catheter just above the SVC-RA junction, and performing ablation at the SVC-RA junction until entrance block is achieved into the SVC. Afterwards, dissociation of SVC potentials may be seen, and pacing from the multi-electrode catheter may capture the SVC myocardial sleeve with exit block into the RA **(Figure 1)**. Ablation may be performed using either a segmental or circumferential approach. With segmental ablation, the earliest signals are targeted sequentially until block is achieved. Circumferential ablation, in contrast, typically involves sequentially connecting lesions until the SVC is entirely circumscribed **(Figure 2)**. The majority of SVC muscular sleeves are discontinuous and heterogeneous, with a thinner or absent sleeve at the posterior wall, which corresponds to an area of low or absent voltage on electroanatomic mapping.^2,6,22^

In addition, the SVC-RA junction is a complicated anatomical area with multiple structures located in close proximity, including the right superior pulmonary vein (RSPV), the right pulmonary artery (RPA), the SAN, and the right phrenic nerve. Because of this, the segmental approach may better minimize the risk of collateral damage because of its more limited ablation effect compared with empiric circumferential ablation.

**Figure 3:**
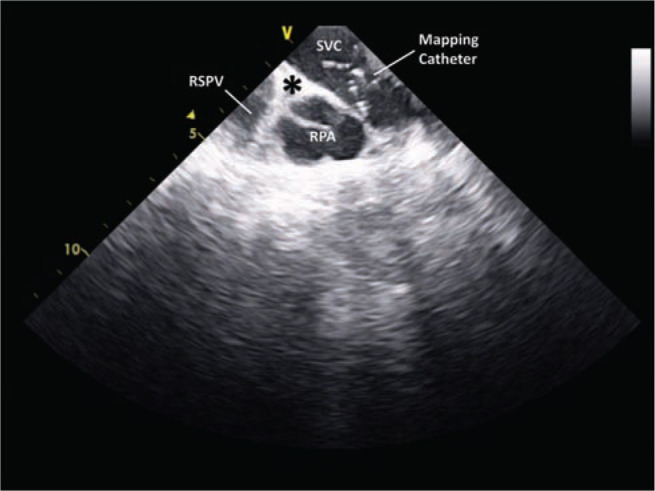
The superior vena cava-right atrium (SVC-RA) junction (marked by an asterisk) at the inferior border of the right pulmonary artery (RPA) on intracardiac echocardiography. The right superior pulmonary vein can be seen inferiorly to the RPA. A multi-electrode mapping catheter is present in the SVC, just above the AVC-RA junction.

Identifying the SVC-RA junction can be done by creating a geometry using electroanatomic mapping, using intra-cardiac echocardiography (ICE) and/or by documenting double potentials consisting of far-field atrial potentials and SVC potentials **(Figure 1)**.^23^ One ICE, the SVC-RA junction is defined by the lower right pulmonary artery border **(Figure 3)**.^15,^^17,18^ Ablation should be performed slightly above the SVC-RA border in order to avoid the sinus node, which is usually located at the anterolateral SVC-RA junction **(Figure 2)**, and only after phrenic nerve capture is excluded.

It is worth noting that the RSPV and the SVC are in such close proximity to each other that foci originating from one may be confused as that originating from the other **(Figure 2)**. In fact, cryoballoon ablation of the RSPVin the majority of patients results in a delay in the conduction of SVC potentials.^24^ Arruda et al. have also suggested that a myocardial connection may exist between the RSPV and SVC, and noted in their study that all patients with SVC triggers also had trigger activity from the adjacent RSPV. In these individuals, PVI alone failed to terminate atrial tachycardia or AF, and only successful SVCI was found to be able to achieve the elimination of arrythmia.^15^ This led Corrado et al. to postulate that SVC isolation may actually complete RSPV isolation.^18^ The placement of a mapping catheter in the RSPV while mapping the SVC can confirm the site of origin and reduce the risk of misclassification of signals.

### Preventing complications from SVCI

Potential complications of SVC isolation include phrenic nerve injury, sinus node dysfunction, and stenosis of the SVC.

The most common complication reported from SVC isolation is right phrenic nerve injury. The right phrenic nerve usually runs posterior and lateral to the SVC and RA, and descends anteriorly to the RSPV and lung hilum, before touching down on the right diaphragm.^25^ The incidence of phrenic nerve injury is low, ranging from 0% to 5%, and is frequently transient, with full recovery.^11,18,25^ The most common way to reduce the risk of phrenic nerve injury is to perform high-output pacing prior to ablation. If diaphragmatic stimulation is seen, which most commonly occurs at the posterolateral SVC if at all, ablation has traditionally been avoided. Arruda et al. noted that a pacing output of 30 mA was required to exclude diaphragmatic stimulation, with a lower output of 10 mA being insufficient, and resulting in a 11% transient phrenic nerve injury with ablation.^15^ Ichihara et al. demonstrated that adequate contact force is also necessary for proper phrenic localization, finding that insufficient contact force (i.e. <10 g) can lead to under-recognition of potential diaphragmatic capture.^26^ The proximity of the phrenic nerve limits SVC isolation in 13% to 18% of patients.^15,18^

More recently, emerging techniques may allow for safe ablation at sites of phrenic capture. Monitoring right diaphragmatic compound motor action potentials (CMAPs), as is used with cryoballoon ablation, can be used to anticipate and prevent phrenic nerve injury, even with phrenic nerve capture.^27^ At SVC sites where the phrenic nerve is captured with high-output pacing, radiofrequency ablation (RFA) can be delivered while pacing the right phrenic nerve from the right subclavian vein. Ablation should be terminated immediately, however, if there is a reduction in strength of diaphragmatic contraction, or a > 30% reduction of the maximum baseline CMAP amplitude. Using this technique, Miyazaki et al. successfully performed RFA at sites of phrenic nerve capture without phrenic nerve injury in 98.6% of radiofrequency (RF) applications.^27^ In one application (1.4%), RF application was interrupted due to a decrease in CMAP amplitude, without evidence of phrenic nerve injury, and subsequent recovery of CMAP amplitude.^27^

SVC stenosis is another common concern with SVCI, but clinically significant stenosis is quite rare. Fenelon et al. raised awareness of this potential complication by demonstrating that RFA in canine caval veins was associated with luminal narrowing in 100% of the cases examined, and was moderate or severe in 50%.^28^ However, in clinical practice, there is a very low incidence of clinically significant SVC stenosis. Callans et al. described that RFA in the SVC of humans for inappropriate sinus tachycardia resulted in an average acute luminal narrowing of 24%, with some lesions associated with small thrombi. However, no patients developed clinical symptoms of SVC syndrome, nor did the three patients who presented for repeat ablation demonstrate any evidence of SVC stenosis.^29^ There has been one reported case of SVC stenosis secondary to circumferential ablation of the SVC for atrial fibrillation, in which the patient had no clinical sequelae on follow-up.^30^ Strategies to reduce the risk of SVC stenosis include using low power during ablation, performing cryoablation instead of RFA, and/or adopting a segmental isolation strategy over circumferential ablation.^30^

Sinus node injury may also complicate SVCI, but, again, this is quite rare. The upper border of the sinus node is located at the anterolateral SVC-RA junction, and, consequently, ablation should be performed slightly above the junction and slightly into the SVC **(Figure 2)**. Chen et al. documented sinus node injury in 4.5% (six of 132) of patients undergoing SVCI who were all transient cases, except for one that required permanent pacemaker implantation.^23^ They acknowledged sinus node injury as a rare complication of SVCI, and partially attributed their higher incidence of complications to reliance on SVC angiography to define the SVC-RA junction. Owing to rapid regurgitation of contrast, SVC angiography may not accurately define the junction. Consequently, proposed strategies to minimize the risk of sinus node injury include using alternative modalities to define the SVC-RA junction (such as ICE and/or electroanatomic mapping); identifying the sinus node with high density mapping; and empirically ablating slightly higher on the anterolateral wall of the SVC compared with the septal wall.

### Persistent left-sided SVC and anomalous PV drainage

A persistent left-sided SVC (PLSVC) is the most common congenital venous anomaly in the chest, and drains the left brachiocephalic trunk into the coronary sinus.

During development, it typically becomes atretic and forms the ligament of Marshall, which is known to be arrhythmogenic.^2^ The incidence of a PLSVC ranges from 0.1% to 3%, and the incidence of a sole PLSVC without a corresponding right-sided SVC is 0.1%.^31^ There are several reported cases of ablating triggers arising from the PLSVC, with most originating at the junction of the PLSVC and coronary sinus.^31–34^ PLSVCs are arrhythmogenic, secondary to multiple electrical communications with the atria that can generate rapid repetitive discharges that may trigger or perpetuate AF.^35^ Ablation of the triggers in one case resulted in freedom from AF for 12 months.^33^ Complications to be wary of include left phrenic injury, SVC stenosis, and/or damage to the left circumflex artery.^31^

Anomalous pulmonary veins, in which the pulmonary veins drain into chambers other than the LA, are rare, with an incidence of only 0.5%.^36^ In the literature, most cases of an anomalous pulmonary vein associated with AF involve an anomalous RSPV draining into the SVC.^37–39^ In all cases, no triggers or potentials could be found in the anomalous pulmonary veins. However, myocardial sleeve potentials were still present within the SVC. Histopathologic examinations have supported this finding, demonstrating that myocardialization of the pulmonary vein is dependent on a normal, open connection to the LA, explaining why no myocardium is seen covering anomalous pulmonary veins.^40^ Although there may not be a need for isolation of the anomalous pulmonary veins in these individuals, SVCI should still be considered. Increased left-to-right shunting, secondary to an anomalous pulmonary vein, contributes to elevated right-sided pressures and, therefore, increased right-sided myocardial stretch and strain. Case reports of three patients with AF and an anomalous RSPV to the SVC all demonstrated significant SVC sleeve potentials, which, following ablation, resulted in a period of freedom from arrhythmia ranging from 5 to 12 months.^37–39^

## Conclusions

Non-pulmonary vein triggers have been identified as a potential source for AF, with SVC triggers among the most common. Ablation of SVC triggers can prevent AF recurrence. When performed in conjunction with PVI, it can also improve outcomes, especially in paroxysmal atrial fibrillation. However, SVC triggers are less common in persistent atrial fibrillation, and empiric SVC ablation is not necessary in this population. At this point, SVCI should be reserved for patients with paroxysmal AF with demonstrable SVC triggers.
